# RETRACTED: Atta et al. Preparation of pH Responsive Polystyrene and Polyvinyl Pyridine Nanospheres Stabilized by Mickering Microgel Emulsions. *Nanomaterials* 2019, *9*, 1693

**DOI:** 10.3390/nano16120754

**Published:** 2026-06-16

**Authors:** Ayman M. Atta, Abdelrahman O. Ezzat, Hamad A. Al-Lohedan, Ahmed M. Tawfeek, Abdulaziz A. Alobaidi

**Affiliations:** Surfactant Research Chair, Chemistry Department, College of Science, King Saud University, P.O. Box 2455, Riyadh 11451, Saudi Arabia; ao_ezzat@yahoo.com (A.O.E.); hlohedan@ksu.edu.sa (H.A.A.-L.); atawfik@ksu.edu.sa (A.M.T.); aezzat@ksu.edu.sa (A.A.A.)

The journal retracts the article titled “Preparation of pH Responsive Polystyrene and Polyvinyl Pyridine Nanospheres Stabilized by Mickering Microgel Emulsions” [1], cited above.

Following publication, concerns were brought to the attention of the Editorial Office regarding the integrity of the figures presented in this publication [1].

In accordance with the journal’s standard procedures, the Editorial Office and Editorial Board conducted an investigation that identified indications of inappropriate image editing and duplication in panels presented in Figures 1, 3, 6, 7, and 9, including material overlapping with previously published articles [2,3]. Although the authors cooperated with the investigation and provided supporting materials, the Editorial Board concluded that the concerns could not be satisfactorily resolved based on the available information. As a consequence, the Editorial Board lost confidence in the validity of the images and has decided to retract this publication in accordance with MDPI’s retraction policy.

This retraction was approved by the Editor-in-Chief of the journal *Nanomaterials*.

The authors have been informed of this retraction, but did not provide a comment on this decision.
